# Pharmaceutical and drug delivery applications of pectin and its modified nanocomposites

**DOI:** 10.1016/j.heliyon.2022.e10654

**Published:** 2022-09-16

**Authors:** Welela Meka Kedir, Ebisa Mirete Deresa, Tamiru Fayisa Diriba

**Affiliations:** a Department of Chemistry, College of Natural and Computational Sciences, Mattu University, Mattu, Ethiopia; b Department of Chemistry, College of Natural Sciences, Jimma University, Jimma, Ethiopia

**Keywords:** Pectin, Polysaccharide, Nanocomposites, Pharmaceuticals, Drug delivery

## Abstract

Due to their natural availability, biocompatibility, biodegradability, nontoxicity, flexibility, as well as improved structural and functional characteristics, pectin and pectin-based nanocomposites have become an interesting area of numerous researchers. Pectin is a polysaccharide that comes from plants and is used in a variety of products. The significance of pectin polysaccharide and its modified nanocomposites in a number of applications has been shown in numerous reviews. On their uses in pharmaceutical and medication delivery, there are, however, few review publications. The majority of papers on pectin polysaccharide do not structure their explanations of drug distribution and medicinal application. The biological application of pectin nanocomposite is also explained in this review, along with a recent publication. As a result, the goal of this review was in-depth analysis to summarize biological application of pectin and its modified nanocomposites. Due to their exceptional physicochemical and biological characteristics, pectin and its nanocomposites are remarkable materials for medicinal applications. In addition to enhancing the immune system, controlling blood cholesterol, and other things, they have been shown to have anticancer, antidiabetic, antioxidant, anti-inflammatory, immunomodulatory, and antibacterial properties. Because of their biocompatibility and properties that allow for regulated release, they have also received a lot of interest as drug carriers in targeted drug delivery systems. They have been used to administer medications to treat cancer, inflammation, pain, Alzheimer's, bacteria, and relax muscles. This review found that pectin and its derivatives have better drug delivery efficiency and are viable candidates for a wide range of medicinal applications. It has been advised to conduct further research on the subject of toxicity in order to produce commercial formulations that can serve as both therapeutic agents and drug carriers.

## 1 Introduction

Because of their abundance and natural availability, biopolymers are currently receiving a lot of interest in the food and pharmaceutical industries [1, 2]. Due to its intrinsic qualities like as biocompatibility, biodegradability, nontoxicity, flexibility, and improved structural and functional aspects, bio-nanocomposites have become the topic of substantial research [3, 4, 5, 6]. Pectin, chitosan, alginate, cellulose, agarose, guar gum, agar, carrageenan, and gelatin are examples of biopolymers and currently hot area research [4, 5]. Biopolymers are particularly intriguing because they are renewable, have a cheap production cost, and have a wide range of pharmaceutical applications [4, 6]. There are three main classes of biopolymers owing to their universal occurrence and abundance: (i) polynucleotides, (ii) polypeptides/poly amino acids, and (iii) polysaccharides [7, 8].

Pectin is a polysaccharide found in plants' cell walls that helps them to grow and extend their cells [5, 9]. Pectin is derived from plants and can be utilized as a bioplastic material for a range of applications [10, 11]. It is a carbohydrate polymer derived mostly from natural sources that serves as a structural component of plants' cell walls [12, 13]. Pectin is a biocompatible polysaccharide with biological activity that can take on many shapes depending on the source or extraction method [14, 15]. It is a poly α 1–4-galacturonic acid containing carboxylic acid residues that have been methylated to various degrees [8, 16]. The most critical parameter affecting pectin's solubility and gel forming characteristics is the degree of esterification of galacturonic acid residues [14, 17]. Since it has low cost, biodegradability, water solubility, and non-toxicity, pectin can be utilized for a variety of reasons [16, 17]. Due to outstanding thermal, mechanical, and biodegradable qualities, biopolymer-based nanocomposites including pectin nanocomposites have attracted a lot of attention in recent years [18, 19]. Pectin is employed in a number of pharmaceutical, cosmetic, food, and biological applications due to its biocompatibility, biodegradability, and non-toxicity [20]. In addition, pectin based bio nanocomposites have various applications in tissue engineering [21], gene transfer, wound healing, and dressings [22, 23], drug delivery [24], and cancer targeting [25]. In the cosmetics sector, it is utilized as an emulsifier. In oral formulations for drug administration to the colon, it is frequently employed in conjunction with kaolin [21]. Furthermore pectin are used to form edible films, and plasticizers [10, 11]. Pectin can have a range of structures depending on the source and extraction procedure [26, 27]. Numerous studies and review reports have demonstrated that pectin and its modified nanocomposites (NCs) are used for a variety of applications. However, there are a little number of reports on their pharmaceutical and drug delivery applications. Therefore, the aim of this review is to compile reports on the pharmaceutical applications of pectin and its modified NCs.

## 2 The review methodology

### 2.1 The study design

A comprehensive review study design were adopted to gather the general information regarding pectin polysaccharide and its modified NCs, including pharmacological and DDS applications, was assembled in this review.

### 2.2 The search strategy

The relevant sources were retrieved by using search engines such as Google scholar and PubMed. Particular keywords that helps to search related study phrases and synonyms such as biopolymer, pectin, polymer nanocomposite, pharmacological use/drug delivery application, biological properties and functionalization of polysaccharide, modification of biopolymer etc. were adopted.

### 2.3 Inclusion and exclusion criteria

Studies reporting pharmaceutical and/or drug delivery application of pectin and its modified NCs were as the other reports related biopolymer were excluded in this review. Studies published in languages other than English were omitted.

### 2.4 Study selection

A short study of the topics, abstracts, and conclusions of the sources was done to determine their eligibility after a relevant sources that helps to the review were gathering. The published paper/review article used as sources in these review were carefully examined in order to prepare this review paper.

### 2.5 Software used

Mendeley Desktop reference management software was utilized to generate references and citations for this review.

## 3 Pharmaceutical application of pectin and structurally modified pectin

Pectin and structurally modified pectin are the most promising pharmaceutical and medicinal applications as well as used for drug delivery system (DDS) [28, 29]. Increasing interest in pectin is due to its easy availability in nature and increasing availability in the pharmaceutical industry [30]. Due to their exceptional physical, chemical, and biological capabilities, biotechnologists and microbiologists have developed various types of biopolymers for specific applications in the biomedical and pharmaceutical industries [31, 32]. These functions make pectin biopolymer a noteworthy product for pharmaceutical and biological applications [26, 33]. The pharmacological properties of pectin and its modified NCs, which include anticancer, antidiabetic, antioxidant, anti-inflammatory, antibacterial, immune system strengthening, and blood cholesterol regulating properties, are discussed in the following section.

### 3.1 Pectin in the cancer treatment

The incidence of cancer is increasing owing to metastases and tumor cell medication tolerance, even though a wide range of scientific investigations have been stepped up to tackle this disease [25, 34]. Pectin has been shown in studies to play a role in the prevention of metastasis, which is especially true of pectin that has been broken down into smaller fragments with a lower molecular weight that the body can absorb [25, 34]. Pectin that has been altered by the use of chemicals, heat, radiation, and enzymes has stronger anticancer properties than unaltered pectin [26, 35]. The proliferation and migrating of colon carcinoma cells have been shown to be inhibited by functionally engineered pectin that contains neutral sugar sequences with a low degree of branching and is rich in galactose [36].

### 3.2 Pectin in the regulation of blood cholesterol level

Highly viscous pectin can have a greater impact on inhibiting the blood cholesterol level by disrupting the micelle formation, slowing down bile acid diffusion, blocking the absorption of micelles carrying cholesterol, and reducing bile acid diffusion rates [37, 38]. The cholesterol-lowering properties of citrus peels are likely due to pectin from the peels [39]. Without making any dietary changes, pectin consumption of at least 6 g/day can lower cholesterol levels in people with normal or elevated lipid levels, lowering the risk of coronary heart disease [37, 40]. The level of plasma triglycerides remained unaltered. Pectin from prickly pears has been found to affect guinea pigs' hepatic cholesterol homeostasis. Hepatic cholesterol homeostasis has been reported in guinea pigs to be altered by intake of pectin from prickly pear [37, 38]. The capacity of pectin varieties utilized to generate a viscous gastro-intestinal content, which is demonstrated to influence substantially the precise molecular composition, is proven to have a significant impact on the lowering of cholesterol and may also affect blood glucose levels [41].

### 3.3 Antioxidant activity

**Figure 1:**
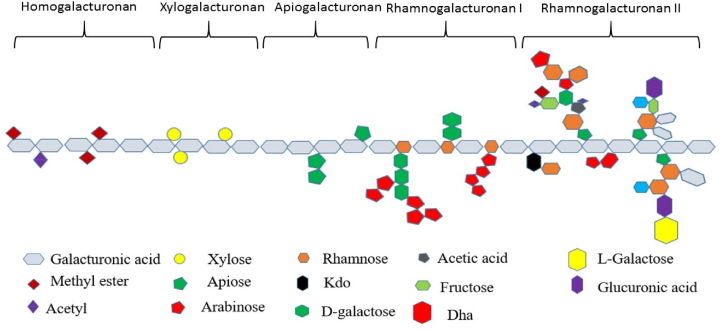
Structural characteristics of pectin molecules. There are different structural characteristics of pectin molecules including Homogalacturonan, Xylogalacturonan, Apiogalacturonan, Rhamnogalacturonan I and Rhamnogalacturonan.

Pectin is a reliable antioxidant that possesses the ability to scavenge free radicals and surpass synthetic substances in the subject of health concerns. Pectin RG-I (rhamnogalacturonan I) exhibited good antioxidant activity as measured by the capture of DPPH- and ABTS- + radicals. Pectin having long HG-1 (homogalacturonan) segments alternated with RG-I segments, arabinogalactan type I and arabinanas side chains (Figure 1) has been found to provide effective protection against the oxidative action of intestinal stress [17]. The hydroxyl groups of polysaccharides in pectin can show good antioxidant activity when the viscosity is not too high [42]. The ferric reducing ability of plasma (FRAP) assay and DPPH scavenging activity [43]. The nano-scaled Fe_3_O_4_ with pectin nanoparticles had effective antioxidant activity based on the IC50 value. Recent nanoparticles appear to have an anti-human liver cancer effect due to their antioxidant properties [44].

### 3.4 Antidiabetic activity

Diabetes mellitus is a primary metabolic condition affecting 382 million people globally. The most common form of diabetes, type 2 diabetes mellitus, affects over 90% of people with the disease [45, 46]. Despite being often used to treat diabetes, artificial antidiabetic drugs have the potential to have negative side effects. As a result, natural compounds have gained a lot of attention in the fight against diabetes [45]. Citrus pectin has potential antidiabetic properties, which were studied in diabetic rats and effective in treating type 2 diabetes mellitus brought on by a modest dose of streptozotocin and a high-fat diet. It's also been demonstrated that citrus pectin improves hyperlipidemia, hepatic glycogen content, and glucose tolerance in diabetic rats [17]. According to earlier research [47], methoxylated apple pectin might be used as a functional ingredient to lower insulin resistance. Furthermore, soybean pectin improved glucose and insulin response in people of normal weight [46]. Based on earlier reported results, it is estimated that pectin and modified pectin have the potential to reduce blood sugar levels and insulin resistance.

### 3.5 Antimicrobial activity

*S. aureus* and *E. coli* are both significantly resistant to the antibacterial effects of pectin. The capacity of pectin-linoleate and pectin-oleate to 50–70% inhibit the growth of the selected bacteria was demonstrated. They have the greatest antibacterial power against *S. aureus* [42]. Additionally, pectin exhibits strong antibacterial activity against various strains of clinically isolated Helicobacter pylori [48]. Additionally, intriguing antibacterial activity has been seen in nanoparticles and nanocomposites that used pectin as the reducing and capping agent throughout production. The antibacterial activity of pectin-Ag NPs was also revealed against *S. aureus* and *E. coli*. Pectin-cadmium sulfide nanocomposite and pectin-based zirconium (IV) silicophosphate nanocomposite (Pc/ZrSPNC) were both found to exhibit strong antimicrobial activity against *E. coli* and *S. aureus*, respectively [17]. The antimicrobial and antiviral activities of pectin and structurally modified pectin have been the subject of numerous investigations, which are compiled in Table 1.

**Table 1:** Antimicrobial activity of pectin based NCs.

Pectin and its nanocomposites	Types of microbes	Microbial strains	Reference
Pectin-oleate, pectin-linoleate, and pectin palmitate	Bacteria	*S. aureus* and *E. coli*	[17]
Ag NPs using pectin as the reducing and capping			
Pectin-based zirconium (IV) silicophosphate NCs			
Pectin–CdS NCs		*E. coli*	
pectin	Bacteria	*H. pylori*	[48]
Pectin-Amphotericin B imine and amine conjugates	Fungus	*C. albicans and A. fumigatus*	[49]
Pectin	Fungus	*Colletotrichum gloeosporioides, Fusarium oxysporum, Sclerotinia sclerotiorum and Mucor sp.*	[50]
Pectin	Fungus	*C. albicans* and *S. cerevisiae*	[51]
Pectin	Virus	Herpesvirus type 1 (HSV -1) poliovirus strain (PV).	[52]
Pectin methylesterase	Virus	Tobacco Mosaic Virus	[53]
Pectin	Virus	Hepatitis B Virus	
Pectin	Virus	Herpes Simplex Virus Type 2	
Pectin	Virus	SARS-CoV-2	

### 3.6 Anti-inflammatory and immunomodulatory activity

As models of inflammation, endotoxin shock, acetic acid-induced colitis, and blood leukocyte production of cytokines in response to lipopolysaccharide have all been used [54]. Intestinal inflammation can be reduced by low methyl-esterified pectin, but systemic and local inflammation can be diminished by high methyl esterified pectin [54, 55]. It has been shown that pectin from *S. dendroideum* leaves affects the release of pro- and anti-inflammatory cytokines by macrophages [17]. Citrus pectin inhibits chemotaxis and phagocytosis, the two main inflammatory processes of chicken monocytes, pointing to potential anti-inflammatory effects [56]. The immunologically stimulating effects of pectin may be due to these components [57]. Pectin were created from an aqueous extract of mulberry fruits that showed immune modulatory activity by improving macrophage function [58]. Furthermore, Rubus chingii Hu pectin can be utilized as a dietary supplement for the treatment of intestinal inflammation and also has a strong inhibitory action on the mRNA level [55]. Pectin polysaccharides exhibit good anti-inflammatory activity and can be improved for the production of anti-inflammatory agents [55, 58]. Further pharmaceutical applications of pectin and its modified NCs were summarized in Table 2.

**Table 2:** Pharmaceutical applications of pectin and structurally modified pectin.

Types of pectin	Sources of pectin	Pharmaceutical activity	Results	References
P/guar ​gum-ZnO	*Cyamposis tetragonotobus* (Seed)	Immuno-stimulator	• The result of the study revealed that the Immuno-stimulatory properties and their increase with the increase in concentration (25–200 μg/mL).	[59]
Pectin	*Sambuci flos*		• Directly stimulate macrophages' immunological systems, encourage cytokine synthesis, and modulate the immune system on several levels.	[60]
Pectin	Citrus ​pectin (Commercial)	Treatment ​of neurological diseases	• In vitro, human neuronal SH-SY5Y cells treated with aqueous H_2_O_2_, a potent oxidant implicated in the cellular pathways causing neurodegenerative diseases, showed significant in vitro neuroprotective and hepatoprotective effects.	[61]
Fe_3_O_4_@p- NPs	Orange peel	Antioxidant and ​anti-liver cancer	• Effectively eliminate by MTT assay the cancer cell lines. • Ability to prevent human liver cancer may be a result of its antioxidant activities (IC50).	[44]
Fe_3_O_4_/P NPs	Apple pomace		• The Fe_3_O_4_/P NPs had the greater antioxidant properties revealed by DPPH assay and the IC50 of Fe_3_O_4_/P NPs was 317, 337, 187, and 300 mg/mL.	[62]
P/Ag ​and ​p/Au–Ag ​NCs	Citrus ​pectin (Commercial)		• Pec/FA/Au–Ag NCs has shown strong radical scavenging activity on DPPH assay.	[25]
P/Tannic acid NCs	Apple pomace and citrus fruits	Anticancer activity	• Pectin drugs NCs are much more effective against PDAC cells Compared to free drug. • Anticancer activities against pancreatic cancer cells which was supported by cell viability and clonogenic formation.	[63]
P/Guar ​Gum/Zinc ​Oxide ​NCs	Commercial Citrus pectin		• The cytotoxicity assay demonstrated that the Pec-GG-ZnO was a greater potential anti-cancer. • Cell cycle study of A549 cells treated with P/gg-ZnO revealed that the S-phase arrest and apoptosis induction. • The mitochondrial depolarization, ROS production, activation of caspase-3, and PARP1 therefore it might be used as anti-cancer therapeutic activity.	[64]
P/Guar ​Gum NCs	Citrus fruit peel	Control plasma hyperglycemia and ​hypercholesterolemia	• Its lower blood sugar, cholesterol, and triglyceride levels.	[65]
Pectin	*Passiflora glandulosa* cav	Reduce blood glucose	• The 200 mg/kg dose decreased blood glucose levels while causing no liver or renal damage in mice. • It has a potential glycemic prevention due to its chemical structure and capacity to gel by lowering glucose absorption.	[66]
Ginseng pectin (GP)	Citrus ​pectin (Commercial)	Neuroprotective effects	• Prevents the cell death brought by hydrogen peroxide in neuronal cells. • Defends the neurites of cortical neuronal cells against deterioration. • Activation of the ERK/MAPK and Akt survival signaling pathways to protect certain neuronal cells from the neurotoxicity caused by hydrogen peroxide (H_2_O_2_).	[67]
Pectin	Citrus ​pectin (Commercial)		• Pectin (4μg) showed the best neuroprotective effects as to preventing neurological impairments and brain edema at 24–48 h post-SAH. • Inhibits galectin-3, whose processes may include binding to TLR4 and activating ERK1/2, STAT-3, and MMP-9, and may prevent post-SAH blood-brain barrier breakdown.	[68]
Pectin	Citrus sinensis (L.) Osbeck peel	Antidiabetic	• Better walking ability, CSL-OP reversed the lowered body weight and elevated blood glucose. • With antioxidant potential, CSL-OP stopped the progression of early diabetic neuropathy. • Pectin therapy at many doses over the course of 4 weeks is effective in slowing or arresting the progression of early diabetic peripheral neuropathy in rats.	[69]
Pectin	Citrus peel		• Both the glucose metabolism and the amount of glucose eliminated by urine were enhanced.	[66]
Pectin	Citrus fruit peel	Glycogen regulation	• Increased brain PKC activity and reduced liver PKC activity, as well as increased glycogenesis and lower glycogenosis. • Increases the serotonin receptor or carrier in the brain and, perhaps by blocking galectin-3, inhibits barrier rupture.	[70]
P/galectin-3 carbohydrate	Citrus ​pectin (Commercial)	Nephroprotective activity	• Control of proliferation, apoptosis, fibrosis, and inflammation, protective in experimental nephropathy. • Prevent long-term kidney damage, possibly through galectin-3's actions related to carbohydrate binding.	[71]
Pectin	Citrus ​pectin (Commercial	Immuno modulatory activity	• Chicken monocytes are subject to CP's immunomodulatory effects, which supports the use of CP in dietary plans that may improve the health and immunity of the animal. • The integration of system biology techniques and inflammatory immune activities may be very helpful in determining the biological importance of CP.	[72]
P/Au ​NCs	Citrus ​pectin (Commercial		• The lower detection limit for insulin by immunoassay is 2.14 pM/L, with a linear range of 50–556 pM/L, an excellent correlation coefficient of 0.98806, good recovery, and great reliability.	[73]
P/ZnO NPs	Pomelo and Citron peel	Antimicrobial activity	• Inhibition zone of the bacteria *B. subtilus* and *S. aureus*, ranged from 15 to 20 mm. • Citrons peel P/ZnO with inhibitory zone widths of 12 mm against *E. coli*. • Only the CPPT-ZnO was considered positive in tumor inhibitions, showing inhibition of 37.09% (» 20%), despite the fact that both nanocomposites were active in tumor inhibitions.	[74]
P/Ag NPs	Citrus pectin (Commercial)		• The P-Ag-NC was efficient antimicrobial activity on *B. subtilus* and *E. coli.*	[75]
Pectin	Citrus ​pectin (Commercial	Hepatoprotective activity	• Stops progression of liver fibrosis by inhibiting galactin-3 and inducing apoptosis of stellate cells. • Pectin attenuate liver fibrosis through an antioxidant effect, inhibition of Gal-3 mediated HSCs activation and induction of apoptosis.	[76]
Pectin	Citrus	Antitumor activity	⁃ Human prostate cancer cells undergo apoptosis when exposed to pectin.	[77]
Pectin nano-Se	Citrus		⁃ Ehrlich ​carcinoma ​cells ​undergo ​apoptosis ​in ​order ​to ​inhibit ​metastasis ​and ​increase ​antioxidant ​activity.	[78]
Pectin	*Lonicera japonica*		⁃ 1 mg/mL dosage, pectin inhibit the enhancement of BxPC-3 and PANC-1 pancreatic cancer cells with inhibitory ratios of 66.7% and 52.1%, respectively.	[79]
Rhamnogalacturonan-I ​(RG-I)	Potato pectin⁃		⁃ Inhibit the growth of HT-29 cell, and a considerable G2/M cell cycle arrest was induced.	[80]
Pectic acid	Apple⁃		⁃ Apoptosis induction, growth inhibition, decreased cell attachment, chromatin fragmentation, and membrane blebbing.	[81]
Rhamnogalacturonan-I ​(RG-I), ​Rhamnogalacturonan-I ​I (RG-II)	Persimmon leaves⁃		⁃ Used to prevent Lung metastasis, and NK cell-mediated cytotoxicity against lymphoma tumor cells is enhanced.	[82]
High-methoxyl Homogalacturonan	*Hippophae rhamnoide*⁃		⁃ Increasing the production of lymphocytes, raising macrophage activity, and encouraging NK and CTL activity.	[83]
Pectin	*Portulaca oleracea L. C*	Antiviral	⁃ Anti-HSV-2 activity	[51]
Pectin	*Saussurea laniceps*		⁃ Prevents ​the ​Hepatitis ​B ​virus ​from ​secreting ​its ​surface ​and ​envelope ​antigens ​in ​HepG2 ​cells.	[84]
Pectin	Inga spp⁃		⁃ Prevent viral replication through binds to the glycoprotein and carboxyl groups on the cell membrane.	[52]
Pectin	*Citreous peel*⁃		⁃ Binding to the protein receptor retard the replication of the SARS-CoV-2.	[85]

Key: P = pectin, NPs = nanoparticles, NCs = nanocomposites.

## 4 Pectin and its modified NCs for drug delivery applications

DDS is a formulation that allows a pharmaceutical agent to reach its target site of action while avoiding non-target cells [21]. In order for a pharmaceutical ingredient's therapeutic agent to be released in a controlled manner, DDS are devices that are meant to carry the substance throughout the body. The active ingredient is less likely to be disrupted physically, chemically, or enzymatically when the molecules are enclosed in a protective shell-like structure. As a result, not only is the active compound's bioavailability increased, but also the adverse effects linked to systemic, non-specific distribution are reduced. The number of dosages needed during therapy is decreased by nano-encapsulating bioactive chemicals, and it's possible that the drug will also be physically protected while being stored before being used for controlled drug release [86]. Natural polymers like pectin have gotten a lot of attention as drug carriers. Because of its biocompatibility, health advantages, nontoxicity, and biodegradability, pectin has been employed to make DDSs [86, 87]. It has a low production cost and is widely available [87]. Protective agents against enzymatic proteolysis have been found in a variety of polymers, including pectin. Because pectin stabilizes polypeptide drugs, they stay intact in the stomach and small intestine before being digested by colonic bacteria, resulting in drug molecule release [88]. Pectin has been employed in a variety of formulations, including hydrogels, films, microspheres, and nanoparticles, to target various medicines [87]. However, pectin's rich hydrophilic functional groups, including the hydroxyl, free carboxyl, and methyl ester groups, result in pectin's significant swelling qualities, limiting its potential use in DDS. Pectin formulations have the potential to expand under physiological conditions, resulting in premature drug release [88]. As a result, researchers have attempted to alter the structure of pectin in order to create pectin-based hybrid and composite materials [86, 88] using various chemical and physical approaches [87].

**Figure 2:**
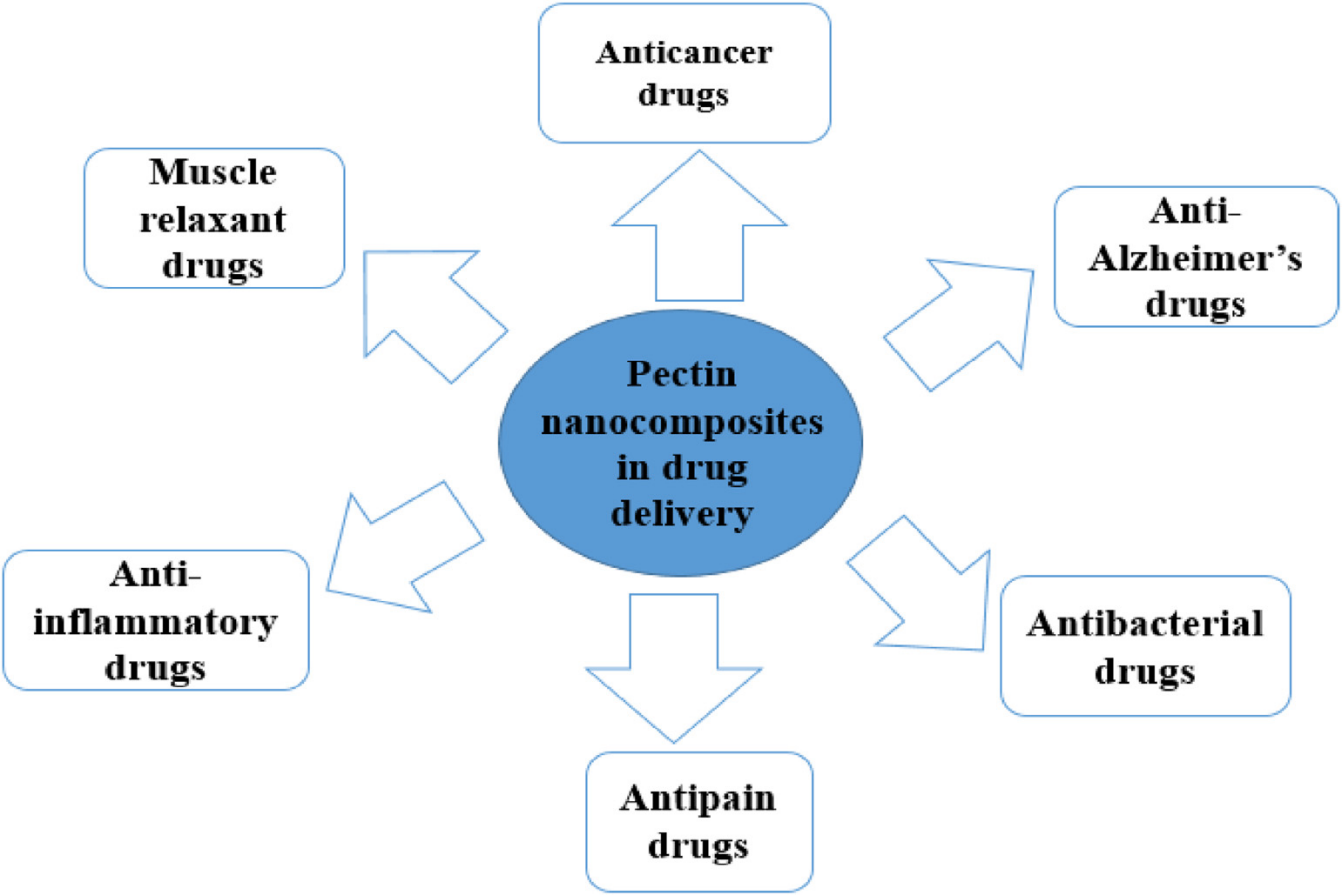
Overview of applications of pectin nanocomposites in drug delivery systems.

Polymer nanoparticles, such as pectin-based nanoparticles, have sparked attention in the biomedical area as gene/DDS due to their biocompatibility and controlled release [24]. Various interactions, such as hydrophobic interaction, electrostatic interaction, and covalent bonding, are commonly used to load medicines into nanoparticles [24, 89]. Drugs or biomolecules can be entrapped within the nanoparticles' internal structures, and/or they can be absorbed onto the nanoparticles' external surfaces [89]. Nanomaterials containing drugs or genes can enter cells by endocytosis rather than diffusion, and because of their small size, they can easily accumulate in target cells. As a result, nanoparticles as a delivery mechanism can reduce drug or gene loss while also increasing delivery efficiency [24]. Furthermore, nanoparticle-based DDS have a number of notable advantages, including the ability to easily pass through the smallest capillary vessels due to their ultra-small volume and avoid rapid clearance by phagocytes, extending their time in the blood stream; the ability to penetrate cells and tissue gaps to reach target organs; they have controlled release qualities [89]. Pectin-based nanomaterials are one strategy of delivering drugs to the colon known as colon targeted DDS. Pectin's swelling properties, as well as its capacity to withstand gastrointestinal degradation, have made it a popular carrier for colon-specific medication delivery. The biodegradability and gel-forming nature of this polysaccharide are the characteristics that drive its selection as a carrier for specific medication delivery [90]. Pectin-based NCs have also been studied for transdermal DDS, in addition to colon-targeted DDS. As shown in Table 3, numerous studies have been undertaken on the application of pectin-based nanocomposites for drug delivery, including anticancer, anti-inflammatory, antipain, anti-alzheimer, antibacterial, muscle relaxant, etc., as depicted in Figure 2.

**Table 3:** Summary of applications of pectin based nanocomposites for delivery of different drugs.

Delivery of anticancer drugs				
Pectin nanocomposites	Delivered drugs	Results		References
Poly ​(acryl-amidoglycolic ​acid-covinyl ​caprolactam)/P/Ag NCs.	5-Fluorouracil	⁃ A 24-hour in vitro study indicated 50% of the 5-fluoro uracil was released by pectin hydrogels at pH 1.2 and released 85% at pH 7.4. ⁃ When the temperature was raised from 25 to 37 °C, the swelling ratio of pectin hydrogels dropped, but was enhanced when the pH was raised from 1.2 to 7.4.		[22]
P/magnetic graphene oxide nanohybrid.	Paclitaxel	⁃ The produced nano-carrier was stable and had a high drug loading capacity. ⁃ Release is larger in cancer cells' endosomal pH than in a physiologically normal environment. ⁃ The nano-hybrid displayed high relative cell viability and biocompatibility in a cytotoxicity test.		[91]
P/tannic acid NCs.	5-fluorouracil, gemcitabine, and irinotecan	⁃ Tannic acid binding enabled the NCs to encapsulate anticancer medicines. ⁃ Internalization for greater therapeutic potential in cellular uptake trials in a dose-dependent way.		[92]
P-nano-cell of core shell structure.	Doxorubicin	⁃ The in vitro anticancer activity result revealed that, it was discovered that doxorubicin accumulated significantly in a variety of tumor cells. ⁃ The doxorubicin-pectin-nano cell improved drug release activity. ⁃ In vitro and in vivo, effective in tumor growth prevention. ⁃ It was also partially reverse drug resistance in tumor cell lines.		[93]
P/Poly (N,N-dimethylacrylamide-stat-4-formylphenyl acrylate) hydrogel	Doxorubicin	⁃ The drug slowly and steadily flowed out of the hydrogels. ⁃ The discharge rate increased as the pH decreased. ⁃ Slow medication release under neutral conditions and increased acidity helped to enhance tumor therapy.		[94]
P-Graft-Copolymers with poly (vinyl alcohol) and their NCs.	5-fluorouracil	⁃ After 300 min in a phosphate buffer solution at pH 7.4 the NCs release was determined to be 93% and 99.1% of 5-fluorouracil respectively.		[95]
P-/lactic acid-co- methacrylic acid hydrogels	Oxaliplatin	⁃ In vitro test of the hydrogels' resistance to lysozyme and collagenase, the hydrogels' stability against a blank phosphate buffer solution was shown to be stronger than that of lysozyme and collagenase. ⁃ The hydrogel dispersion was well tolerated in rabbits up to 3650 mg/kg body weight in the oral tolerance study, with no apparent hematological or histological abnormalities.		[96]
P-Based NPs	5-fluorouracil	⁃ Drug-loading was discovered in 24.8% of the material. ⁃ In vivo pharmacokinetic investigation found that drug-loaded NPs had considerably better bioavailability than free drugs. ⁃ An in vivo bio-distribution investigation in healthy mouse tissue found that NPs had a longer cycle of influence than free medication.		[97]
P-based hollow Nano capsules	Doxorubicin hydrochloride	⁃ After 48 h in bovine serum albumin solution and 96 h in phosphate buffered saline solution. ⁃ The p/chitosan maintained remarkable colloidal stability. ⁃ For doxorubicin hydrochloride, the Nano-capsules displayed significant drug loading and pH-sensitive release.		[98]
P-based magnetic Nanocariers	Oxaliplatin	⁃ The drug loading content and drug encapsulation efficiency of the nano-cariers were determined to be 55.24.8% w/w and 0.100.04 wt. %, respectively. ⁃ The Nano-carriers continuous Oxaliplatin release in phosphate buffer solution at pH 5.5 and 7.4.		[99]
**Delivery of anti-Alzheimer's drugs**				
P/Ag NCs film	Donepezil	⁃ The NCs adsorption and release efficiencies were also exceptional. ⁃ The drug release capacity of the NCs in phosphate buffer saline solution was determined to be 94.33 over a 5-day period. ⁃ It is non-toxic and blood-compatible.	[100]	
P/ZnO hybrid NCs	Donepezil	⁃ The nanocomposite had more drug adsorption (273.91 mg/g) than the parent gel (35.55 mg/g). ⁃ In vitro drug release studies revealed that the NCs increased drug desorption by up to 88% over a 5-day period when compared to the parent gel (46% over a 120-h period). ⁃ The NCs was low toxicity and blood compatible.	[101]	
**Delivery of antibacterial drugs**				
P/polyvinylpyrrolidone,3-aminopropyl (diethoxy) methyl silane and sepiolite clay) hydrogel	Ceftriaxone sodium	⁃ When the pH was reduced, the hydrogels swelled more, suggesting that they are pH-responsive. ⁃ All hydrogels were degraded after 21 days in phosphate buffered saline pH 7.4 (human blood pH). ⁃ In an in vitro cytocompatibility test employing the 3T3 murine fibroblast cell line, the hydrogels were shown to be safe. ⁃ The hydrogel's release profile demonstrated 91.82% release in phosphate buffer saline solution over 2 h and 20 min in a consistent and regulated way.	[102]	
P/Chitosan Polyelectrolyte NCs	Nisin	⁃ *In vitro* release assays revealed that the pH of the nano-capsules altered the release profile of Nisin, with more Nisin released at pH 3 than at pH 6.	[103]	
**Delivery of antipain drugs**				
P-based hydrogels	Ibuprofen	⁃ Based on *in vitro* drug-release experiments in multiple buffer solutions, the release value of Ibuprofen from hydrogels at pH 7.4 was greater than that at pH 1.2.	[104]	
Methylcellulose/p/Montmorillonite NCs films	Ketorolac tromethamine	⁃ *In vitro* transdermal release of Ketorolac tromethamine revealed that the NCs films released the drug constantly over 7 h in phosphate buffered saline media at 37 °C. ⁃ The duration it took for the drug to be released increased as the concentration of montmorillonite was increased from 1 to 5%wt. ⁃ A NCs film coated with 5%wt Montmorillonite performed more effectively in terms of controlled release of a transdermal medicine.	[105]	
**Delivery of anti-inflammatory drugs**				
P-coated chitosan– layered double hydroxide bio- NCs beads	5-aminosalicylic acid	⁃ The NCs was proven to be resistive towards water swelling and obtained a controlled release of the drug along its transit through the simulated gastrointestinal tract in *vitro* tests owing to its resilience to pH variations.	[106]	
P/Zn/Alginate Core-Shell Beads	Betamethasone	⁃ Pectin (4.0% w/v) and an alginate (2.0% w/v) (2:1 core: shell ratio), served as a sustained DD method. ⁃ Low methoxyl pectin core was possible to minimize the medication's imminent preview in the upper region of the gastro-intestinal tract.	[107]	
**Delivery of muscle relaxant drugs**				
P-coated baclofen-layered zinc hydroxide nanohybrid	Baclofen	⁃ *In vitro* drug release tests with pectin encapsulated layered zinc hydroxide-baclofen nano-hybrids revealed that they improved protection towards stomach pH and regulated release under intestinal tract conditions.	[107]	
P/chitosan/Eudragit®RS mixed-film coating	Theophylline	⁃ In formulations with 15 or 20% (m/m) coating mass increase and 5 or 10% (m/m) pectin-chitosan, burst drug release was omitted and replaced by a lag phase of drug release. ⁃ In terms of burst drug release in the colonic medium, formulations with a 20% (m/m) coating mass increase and 15% or 20% (m/m) pectin-chitosan performed better than the other formulations.	[108]	
**Delivery of other drugs/supplements**				
P/Cu-based ​metal–organic framework nanofiber	Folic acid	⁃ Folic acid release from NPs was rapid during the first 44 h of exposure to phosphate buffered solution at 37 °C, then slowed and eventually peaked at 164 h (approximately 7 days). ⁃ Controlled drug release was achieved because of the advantages of delivery nanofiber and folic acid covalent bonding in NPs structure.	[109]	
P/hydroxyethyl methacrylate hydrogel NCs cross-linked with TiO_2_	Vitamin B_12_	⁃ The pH-dependent changes in vitamin B12 release from the hydrogels were observed, and it was discovered that as pH rises, the time needed for release to reach equilibrium increases, suggesting sustained release characteristics. ⁃ The TiO_2_ was cross-linked with vinylated pectin and hydroxyethyl methacrylate hydrogel to minimize the first release when compared to a pure p/hydroxyethyl methacrylate hydrogel, with a reduction of up to 60% noted.	[110]	
P/chitosan hydrogels	Hesperidin	⁃ P/chitosan concentration of 5%: 1% produced a higher entrapment efficiency of 96.5%. ⁃ An in vitro drug release study showed that the formula with the highest pectin concentration in a medium containing 2% rat caecum had the greatest drug release rate of 56%.	[111]	
P/chitosan core–shell NPs	Resveratrol	⁃ The result revealed that 89% particle yield and a loading efficiency of more than 55% allowed them to encapsulate a sizeable amount of resveratrol. ⁃ The drug's release efficiency was pH-dependent, and that drug release could be sequentially controlled by altering the shell thickness. ⁃ The percentage of resveratrol released over time from nanoparticles was higher in acidic or alkaline pH than in neutral pH.	[112]	

Key: P = pectin, NPs = nanoparticles, NCs = nanocomposites.

## 5 Conclusion

Many in vitro and/or in vivo studies have revealed that pectin polysaccharide and pectin-based modified NCs have various pharmaceutical activities such as anticancer, antidiabetic, antioxidant, anti-inflammatory, antimicrobial, immune system boosting, blood cholesterol regulation, and so on. In addition to being utilized as active therapeutic agents, pectin-based nanocomposites have been employed in drug delivery systems, including the delivery of anticancer, anti-inflammatory, antipain, anti-alzheimer, antibacterial, muscle relaxant, and other drugs. According to the findings of this review, pectin and its modified NCs could be potential therapeutic agents and drug carriers in the near future if adequate toxicity studies are conducted to confirm their safety for human cells.

## Declarations

### Author contribution statement

All authors listed have significantly contributed to the development and the writing of this article.

### Funding statement

This research did not receive any specific grant from funding agencies in the public, commercial, or not-for-profit sectors.

### Data availability statement

The data was collected from the internet, journals and books. All the statements taken from all the sources are cited in the proper manner.

### Declaration of interests statement

The authors declare no conflict of interest.

### Additional information

No additional information is available for this paper.
